# Teledermatology Use in Remote Areas of French Guiana: Experience From a Long-Running System

**DOI:** 10.3389/fpubh.2019.00387

**Published:** 2019-12-19

**Authors:** Anne-Laure Messagier, Romain Blaizot, Pierre Couppié, Sophie Delaigue

**Affiliations:** ^1^Dermatology Department, Centre Hospitalier Andrée-Rosemon, Cayenne, French Guiana; ^2^EA 3593 Ecosystèmes Amazoniens et Pathologies Tropicales, University of French Guiana, Cayenne, French Guiana

**Keywords:** telemedicine, teledermatology, French Guiana, remote area, neglected tropical diseases, long-running system

## Abstract

**Introduction:** French Guiana is an overseas region of France on the north coast of South America and is mostly covered by tropical rainforest. Most human settlements are located along the coast while some settlements are scattered across the hinterland. In 2001, the French public health service launched a telemedicine pilot project between the main hospital in Cayenne and remote health centers in French Guiana to tackle healthcare access inequalities. The aim of the present study was to review dermatology cases of the French Guiana telemedicine network to assess the use of telemedicine in dermatology, in order to evaluate its usefulness and propose ways to improve the system.

**Methods:** A retrospective study was conducted on all dermatology cases referred between July 2015 and December 2016 through the French Guiana platform. The Model for Assessment of Telemedicine (MAST) methodology was used as recommended by the European Union.

**Results:** A total of 254 cases were reviewed by dermatologists at Cayenne hospital over the 18-month study period, with a mean of 14 cases per month. All the 16 peripheral health centers used the telemedicine service during the study. In most cases (202/254, 80%), specialists provided a single diagnosis to the referrers. Infectious diseases represented the main reasons for requests (92/202, 46%) including 32% (29/92) of neglected tropical diseases like leprosy and cutaneous leishmaniasis. A total of 39% (100/258) peripheral centers answered the end-users' survey, and more than 85% found the answer delay was fast, the service useful and with an educational benefit. Overall, the accuracy of the diagnosis increased with the quality of the pictures provided, though the latter was good in only 60% (75/125) of the cases. Most patients for whom a teleconsultations has been required (234/254, 92%) have been managed in the peripheral health centers, while referring the patient to Cayenne was necessary for only 20/254 (8%).

**Conclusion:** The telemedicine system in French Guiana appears to be an interesting solution to the lack of specialists and allowed a better access to specialized dermatology care for people living in the remote areas of this region.

## Introduction

French Guiana is a department of 83,534 km^2^ located on the north coast of South America whose area is close to Portugal. Ninety-five percent of the area is covered by rainforest. Most of its 280,000-inhabitants population is settled on the coast. The road network is formed by a main axis linking Brazil to Suriname via coastal cities, the interior of the land is only accessible by plane or by boat. For a substantial part of the population, health care access is a real struggle. In 2001, to alleviate this inequality and ease the access of isolated populations to specialists, the introduction of telemedicine was contemplated, through a partnership between the Cayenne Hospital, the National Center for Space Studies (CNES) and Institute of Medicine and Spatial Physiology (MEDES). The first medical specialties of the project were dermatology, parasitology, and cardiology. Gradually, every single medical specialty of the Cayenne Hospital was represented to make available specialized advice to the 16 Delocalized Centers for Prevention and Care (CDPS) spread all over the remote areas of the territory. In dermatology, since diagnosis is mostly visual, telemedicine use was a good approach. In tropical areas, skin diseases are a common reason for consultation in general practice. Among the most frequent medical conditions are cosmopolitan infectious skin disease but also less known tropical pathologies such as leprosy and cutaneous leishmaniasis for whom early diagnosis and treatment can critically help in limiting disease-related complications. In remote areas, these diagnosis represent a real challenge for non-specialists health-care practitioners.

The main objective of this study was as follows. First, to assessing the quality of service for dermatology telemedicine service performed at the Delocalized Centers for Prevention and Care (CDPS) in French Guiana from July 1, 2015 to December 31, 2016. Second, to evaluate the usefulness of the telemedicine service system for referrer who worked at the Delocalized Centers for Prevention and Care (CDPS) in 2016 and 2017. Third, to propose ways to improve the telemedicine system.

## Materials and Methods

### General Principles

All dermatology cases received between July 1, 2015 and December 31, 2016 were retrospectively studied. Data were collected manually by a resident of the dermatology department in an Excel file from the dermatology requests on the Lotus Notes software (IBM, Armonk, USA). We also conducted a short satisfaction survey of referrers who worked in CDPS between 2016 and 2017. The data collected was then subjected to a descriptive statistical analysis. The following functions were mainly used: average, median, percentage.

### The Telemedicine System in French Guiana

**Figure 1:**
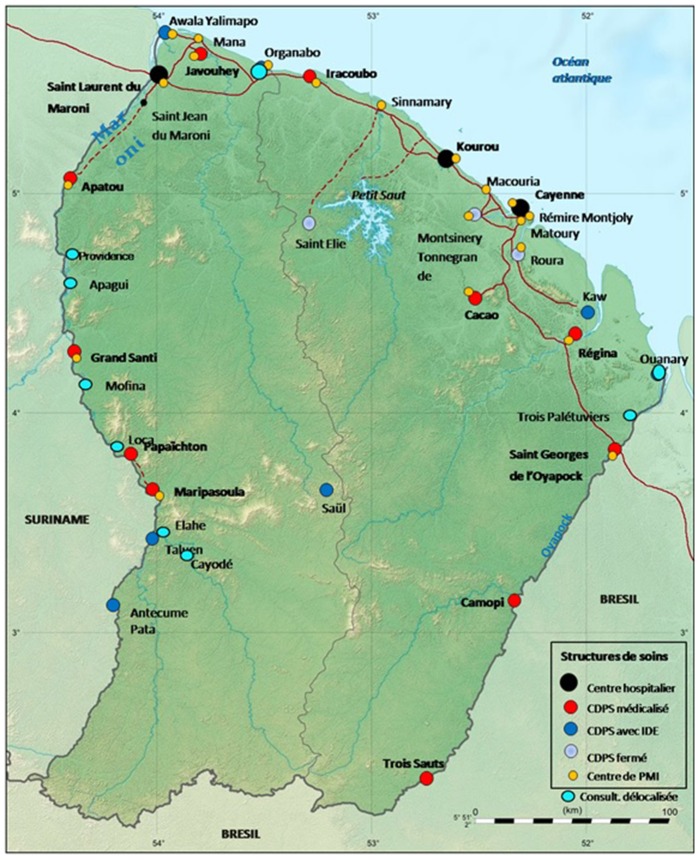
Delocalized Center for Prevention and Care (CDPS and hospitals) in French Guiana.

French Guiana owns three hospitals, all located on the coastal cities of Cayenne, Saint-Laurent du Maroni and Kourou. The rest of the territory is covered by 16 health centers (CDPS) (Figure 1). These centers are administrative subdivisions of the Cayenne Hospital, only eight of them have physicians and medical examination possibility is very limited locally. Dermatologists in French Guiana aren't numerous: four private dermatologists, one dermatologist at the penitentiary center and Cayenne hospital is the only center benefiting from a dermatology department with three dermatologists currently working there. On-field missions to Maripasoula and Saint-Georges are performed by dermatologists of the Cayenne hospital on a monthly basis. They also provide telemedicine consultations for all CDPS in the territory as part of their usual function. Each CDPS is equipped with a computer with access to the telemedicine computer software, they also have a digital camera and internet connection. The request form includes a specific medical observation according to the specialists they reach out to and they can attach additional documents (such as photographs). It is then automatically sent to the specialist according to the request form chosen. The dermatologist based in Cayenne does not receive an alert when a new dermatology request form is sent. They would regularly check the telemedicine platform from the dermatology department, and they answer directly to referrer. Telemedicine is also available in partnership with Martinique for some medical specialty that are not represented in French Guiana, such as neuroimaging and neurosurgery.

### The MAST Evaluation System (Model for Assessment of Telemedicine Applications)

The different evaluation indicators of the French Guiana telemedicine system for dermatology were organized according to the MAST evaluation grid. The MAST methodology is a multidisciplinary approach to evaluating telemedicine projects recommended at European level.

**Table:** 

**Evaluation area**	**Indicators, statistical data**	**Means of verification**
Health problems and characteristics of the demand for care	Total number of cases, average per month (year 2016), number of cases per center	Platform
	Categories of the specialist's answer: (1) Single diagnosis, (2) Several diagnoses, (3) No diagnosis Number of request by diagnostic category (infectious, inflammatory, tumor, genetic, other)	Manual^*^
	Distribution by sex and age	Platform
	Acute (evolution time <3 months) or chronic (>3 months) symptoms	
	Total and average number of photographs per file, file type and size	
Quality and safety	Certainty of diagnosis (Question for the specialists in the response form: 95% certainty of the proposed diagnosis, 75% with a differential diagnosis, 50% with several differential diagnoses, no certainty)	
	Median response time (Time between the date and time of sending the request and the first response of the specialist)	
	Image quality evaluate buy specialist (“good,” “medium,” or “bad”)	
	Clinical details and technical advice (When the specialist felt that there was a lack of patient information or advice on taking photographs, the request was considered incomplete.)	Manual
	Response rate to the survey sent to referrers	Survey to referrers^**^
	Percentage of telemedicine users among responders of the survey	
	Does the average response time seem satisfactory to you?	
	How do you think we could improve this tool? (Free text)	
Clinical effectiveness	Diagnostic Concordance (When the referrer and the specialist proposed a single same diagnosis)	Manual
	Proposed examinations^***^ and treatments (For each request it was noted whether a paraclinical examination or treatment was proposed yes or no by the specialist specifying which type)	
	Does the use of telemedicine allow you to improve your personal knowledge?	Survey to referrers
Patient satisfaction	Do the specialist's recommendations seem to you to be adapted to the resources available on site?	
	Evaluate your satisfaction of the system (Scale from 0 to 10)	
Economic aspect	Patient's outcome (medical evacuation, hospitalization or consultation)	Manual
	Does the use of telemedicine for dermatology seem useful to you? If so, why?	Survey to referrers
Organizational aspect	Number of CDPS that used the platform among those with telemedicine	Platform
	Number of specialists (who answered the requests among the three dermatologists of the service)	
	Number of requests per patient	Manual
	Occupation of referrers (CDPS health professionals)	Platform
	Degree of urgency felt by the referrers	
	Do you give news of the patient after receiving the specialized opinion	Survey to referrers
	If not, why?	
Legal, socio-cultural, and ethical aspects	There is no system for collecting patient consent on the platform	

* *Corresponds to the dermatology residential student in charge of the study*.

** *The list of professionals who worked in the health centers in 2017 was provided by the Cayenne Hospital. The survey was accessible via software hosted on the web: https://www.surveymonkey.com*.

*** *No radiology available on site, only a “descrambling” ultrasound system is available, no delocalized biology only rapid tests (CRP, TROD). Material for microbiological specimens and skin biopsies is available*.

## Results

**Table:** 

**Evaluation area**	**Results**
Health problems and characteristics of the demand for care	**Total number of cases =** 254 requests
	**Mean =** 14.1 cases/month
	**Number of cases per center** (Figure 2)
	**Number of request by diagnostic category:**
	**(1) Single diagnosis 202/254 (80%)**: Table 1 and general classification: - Infectious (pyoderma, leishmaniasis, virosis, dermatophytosis…) 92/202 (46%) - Inflammatories (autoimmune, cutaneous adverse drug reactions, eczema, lichen, psoriasis,…) 85/202 (42%) - Other (e.g., wounds, ulcers, arthropod bites) 21/202 (10%) - Tumor (e.g., lipoma, actinic keratosis, botryomycoma) 3/202 (2%) - Genetics (e.g., neurofibromatosis) 1/202 (1%)
	**(2) Several diagnoses 19/254 (8%)**
	**(3) No diagnosis 33/254 (13%)**
	**Distribution by sex:** Sex -ratio 1,3 (139 men/106 women)
	**Distribution by age [years]: [0–18]** 95/254 (37%), **[18–30]** 52/254 (20%), **[30–60]** 79/254
	(31%), **>60** 22/254 (9%)**, Undetermined** 6/254 (2%)
	**Acute** symptoms: 148/254 (58%), **Chronic:** 77/254 (30%)
	**Total number of photographs:** 888 photographs • **Average:** 3,5 photographs/request • **File type:** JPG 124/254 (49%), PDF 109/254 (43%), JPEG 3/254 (1%), IMG 2/254 (1%), undetermined 16/254 (6%) • **Average size:** 2917 Ko
Quality and safety	**Certainty of diagnosis** - **95% certainty: 42/92 (46%)** - 75% certainty: 12/92 (13%) - 50% certainty: 3/92 (3%), - No certainty 35/92 (38%) - Undetermined 162/254 (64%)
	**Median response time: 1 day and 12 min** (minimum 30 min/maximum 20 days)
	**Image quality**: **Good** 75/125 (60%), **Medium** 35/125 (28%), **Bad** 15/125 (12%), **Undetermined** 129/254 (51%)
	**Clinical details and technical advice:** 64/254 (25%)
	**Response rate to the survey:** 100/258 (39%)
	**Percentage of health professionals using telemedicine:** Yes 71%, No 29%
	**Does the average response time seem satisfactory to you?** Yes 86%, No 14%
	**How do you think we could improve this tool? (Examples of answers)** - **System accessibility:** Simplifying and improving the ergonomics of the software (Importing a photo and sending it takes time). Improve access to other specialties. The opinion request form is too complex and detailed. (Loss of time during consultations). Problem of internet availability. Certain interest in developing the system in private medicine. - **Patient follow** -**up:** Creation of a patient file to guarantee a better follow -up of the patients (rotation of the doctors). - **Training of health professionals in the field:** Training of general practitioners on dermatology to improve sampling, taking photographs. - **Management of patients:** Sometimes it is the availability of medication that is difficult in an isolated environment. - **Financing:** Pricing of telemedicine activity in order to perpetuate the system
Clinical effectiveness	**Diagnostic Concordance** 47/86 (55%)
	**Proposed examinations Biopsies** 44/254 (17%), **Swabs** 13/254 (5%), **Blood test** 22/254 (9%), **Radiography** 2/254 (1%), **Ultrasonography** 5/254 (2%), **None or unspecified** 168/254 (66%)
	**Recommended treatments Surgical excision** 3/254 (1%), **Local treatments** 87/254 (34%), **Systemic treatments** 26/254 (10%), **Local and Systemic** 55/254 (22%), **None or unspecified** 83/254 (33%)
	**Does the use of telemedicine allow you to improve your personal knowledge?** Yes 93%, No 7%
Patient satisfaction	**Do the specialist's recommendations seem to you to be adapted to the resources available on site?** Very good 60%, Moderately 35%, A little 5%, Not at all 0%
	**Evaluate your satisfaction of the system: Average** 7,5/10
Economic aspect	**Patient's outcome Total requiring specialized opinion = 20/254 (8%)** - Sanitary evacuation 2/254 (1%) - Planned hospitalization 5/254 (2%) - Specialized consultation 13/254 (5%)
	**Does the use of telemedicine for dermatology seem useful to you? If so, why?** A lot 90%, Moderately 7%, A little 3%, Not at all 0% - Diagnosis support (100%) - Improvement of patient management (87%) - This reassures you (32%) - Less travel for the patient (58%) - Decrease in health system spending (53%)
Organizational aspect	**Number of establishments users:** 16/16 CDPS
	**Number of specialists Specialist (1)**: 186/254 (73%), **Specialist (2):** 67/254 (26%), **Specialist (3):** 1/254 (0%)
	**Number of requests per patient** - Concerned a single request 237/254 (93%) - Required at least 1 s request for the same patient 17/254 (7%)
	**Occupation of referrer Doctor** 177/254 (70%), **Nurse** 47/254 (18%), **Midwife** 0/254 (0%), **Undetermined** 30/254 (12%)
	**Degree of urgency felt by referrer and examples** - **Very urgent:** 3/190 (2%): a profuse larva migrans infection, a machete -related surinfected deep wound and a cutaneous leishmaniasis - **Urgent:** 46/190 (24%): Severe flare -up psoriasis, “papillonite” - **Not urgent**: 141/190 (74%): Tungiasis, impetigo
	**Do you give news of the patient after receiving the specialized opinion?** Always 11%, Often 23%, Rarely 56%, Not 11%
	**If not, why?** - The patient did not reconsult 48% - I did not have time 27%, - I thought it was not necessary 40% - I did not agree with Answer 4% - I forgot 17% - Other 23%: sample answers ° I would have done in case of adverse evolution (as would a patient) ° Ergonomics of the software not ‘ -adapted to the exchanges ° Short -term replacements, little opportunity to see patients again ° Fear of over -asking specialists ° No request from the specialist

**Table 1:** Examples of the defined diagnostics.

**Standardized diagnosis (number of cases and proportion) Total: 254 (100%)**			
Sore	40 (15.7%)	Cutaneous larva migrans	3 (1.2%)
Pyoderma	22 (8.7%)	Zona	3 (1.2%)
Mycosis	17 (6.7%)	Scabies^*^	4 (1.6%)
Eczema	10 (3.9%)	Leprae^*^	7 (2.8%)
Prurigo	9 (3.5%)	Leishmaniasis^*^	17 (6.7%)
Pso ria sis	6 (2.4%)	Tungiasis^*^	1(0.4%)
Lupus	4 (1.6%)	Yellowtail moth dermatitis^**^	2 (0.8%)
Adverse cutaneous reaction	3 (1.2%)	Others diagnosis	50 (19.7%)
Tinea capitis	4 (1.6%)	Unclear diagnostic	52 (20.5%)

* *Neglected tropical diseases*.

** *Also called Caripito itch or “papillonite” in French Guiana*.

### Health Problems and Characteristics of the Demand for Care

**Figure 2:**
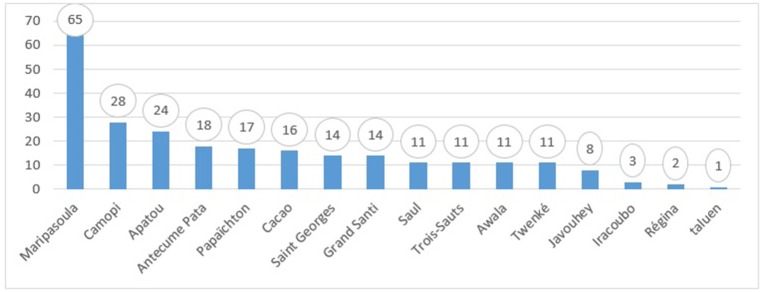
Number of dermatology cases sent to the Cayenne Hospital on the telemedicine platform by peripheral centers between July 1, 2015 and December 31, 2016.

**Figure 3:**
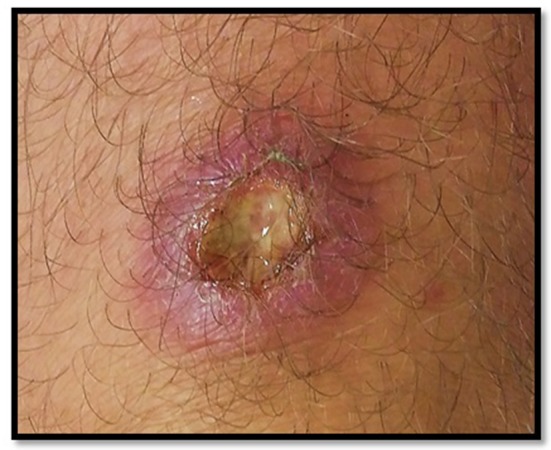
Suspected bacterial infection in a young adult, resistant to antibiotic therapy, considered very urgent by the referral. After expertise: probable leishmaniasis with need to carry out parasitological samples (PCR, cultures) for species identification, then start treatment with Pentamidine.

**Figure 4:**
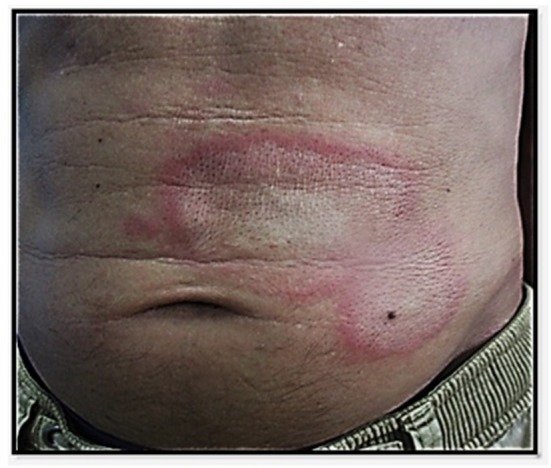
Suspicion of borderline leprosy.

**Figure 5:**
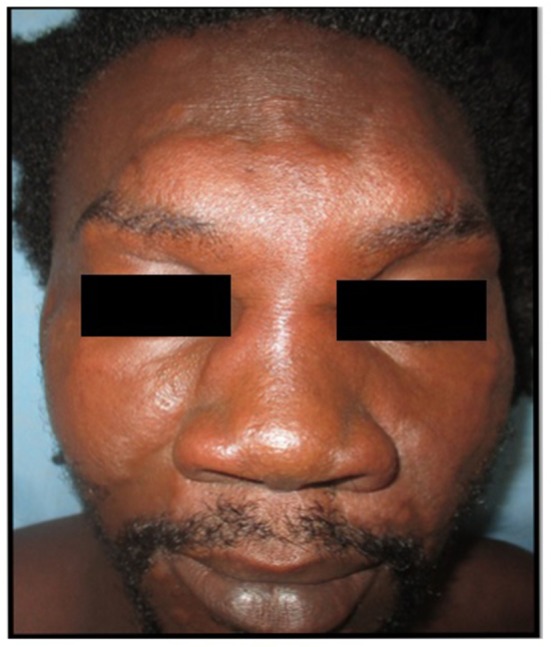
Suspicion of borderline leprosy with type 1 reversal reaction, requiring hospitalization.

**Figure 6:**
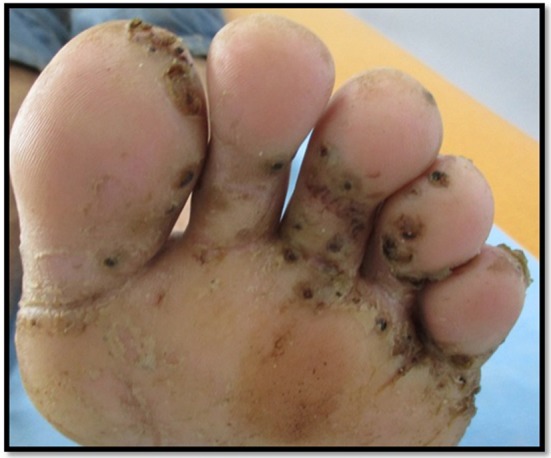
Tungiasis in a febrile patient. Confirmation of diagnosis and introduction of general antibiotic therapy in the peripheral centers (in case of clinical arguments for a dermohypodermitis complication) associated with a manual scalpel extraction of the fleas.

**Figure 7:**
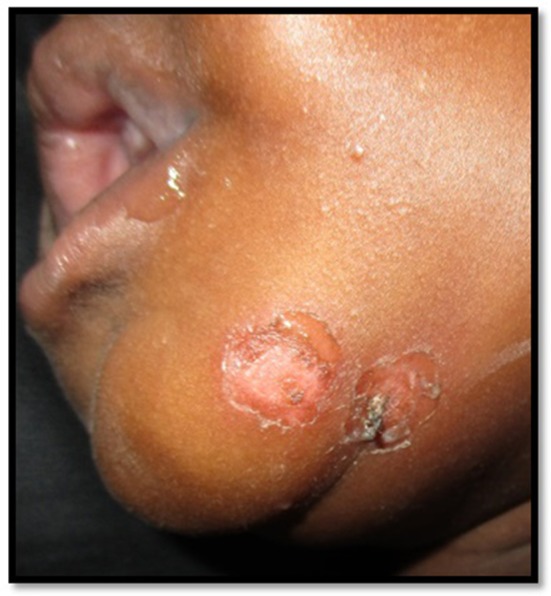
Bullous impetigo in a child, treated in the peripheral center by a general and local antibiotic because of the diffuse impairment.

**Figure 8:**
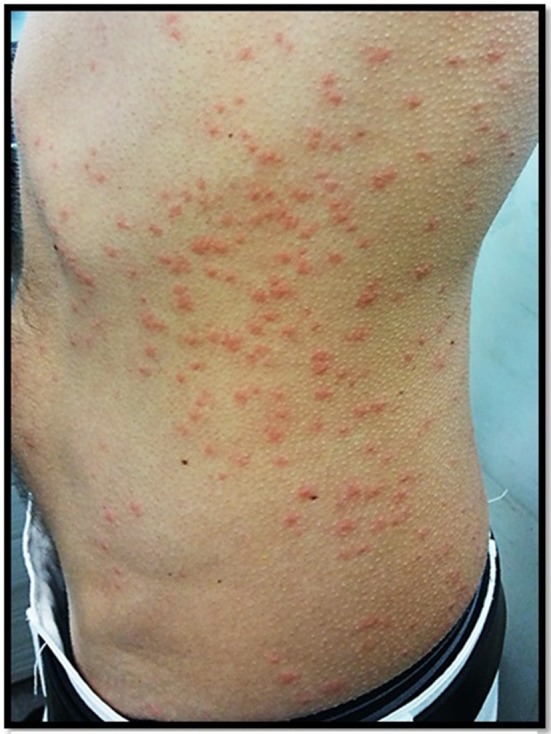
Suspicion of “papillonite” in a patient with a very itchy popular rash of the trunc and limbs.

During the 18 months of study, 254 dermatology cases were analyzed into detail. The Maripasoula CDPS was the lead referrer for dermatology with 26% (65/254) of requests (Figure 2). In their answers, specialists proposed a single diagnosis in 80% (202/254) of cases. In decreasing order of frequency, infectious (92/202, 46%) and then inflammatory (85/202, 42%) dermatoses occurred. Neglected tropical disease accounted for 11% (29/254) of all cases including 17 cases of leishmaniasis and 7 cases of leprosy (three paucibacillary, three multibacillary, one type 1 reversal reaction) diagnosed clinically. Examples of treated cases are illustrated in Figures 3–8. Most cases were acute cases (148/254, 58%), we haven't notice any chronic patients with a follow up of their condition through the platform. On average, requests had more than three photos, and 94% of cases contained at least one image.

### Quality and Safety

**Figure 9:**
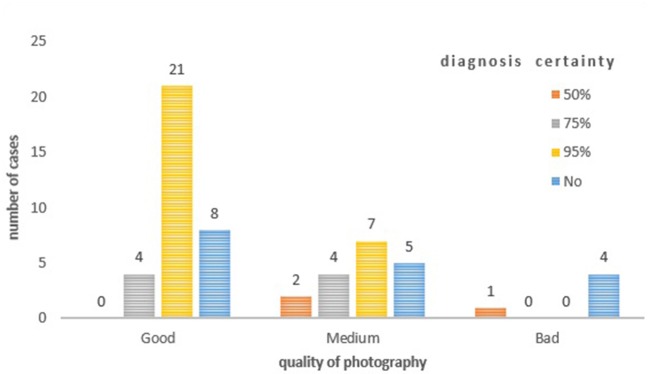
Certainty of diagnosis according to the quality of the photograph.

When filled by specialists, the 95% certainty concerned 46% (42/92) of the answered. The diagnostic certainty was higher when the quality of the photograph was judged to be good by the specialist (Figure 9). There was no difference in diagnostic certainty depending on whether the suspected pathology was inflammatory or infectious. The median time to answer dermatology cases was 1 day and 12 min. Health care practitioners from peripheral centers reported having used telemedicine for 71% of them, and more than 80% are satisfied with the response time.

### Clinical Effectiveness

When the referrer and specialists had proposed a single diagnosis, the concordance rate was 55% corresponding to a similar diagnosis by the two correspondents. Following the diagnosis suspected, the specialist mostly recommended no further examination (168/254, 66%), then a cutaneous biopsy (44/254, 17%). Also, most of cases required a topical treatment (87/254, 34%). According to the satisfaction survey, more than 90% considered teleexpertise as very useful with an improvement in their personal knowledge.

### Economic Aspect

Among the 254 dermatology cases, only 8% (20/254) required face-to-face specialized advice at the expertise center in Cayenne with varying degrees of urgency. Two required emergency medical evacuation (one machete surinfected wound and one deep wound with myiasis). An evacuation involved one of the three patients assessed as very urgent by the referrer. The others required a planned hospitalization or specialized hospital consultation. More than half of the telemedicine users mentioned a decrease in the number of trips for the patient as well as a decrease in health care system expenses thanks to the telemedicine use.

### Organizational Aspect

All the CDPS used the system during the study period. One specialist was mostly involved into the dermatology telemedicine program, as he answered 73% (186/254) of all cases. Referrers from peripheral health centers were mostly physicians (177/254, 70%). The majority of cases were considered non-urgent by the referrer (141/190, 74%). Most patients required a single consultation with one question from the referrer and a single answer from the specialist. Few patients required a follow-up of the case with more questions or a second request at a later stage (17/254, 7%). Generally, the referrers rarely gave (56%) follow-up patient informations to the specialist.

## Discussion

The teledermatology system worked regularly during the 18 months of the study allowing permanent support by the center of expertise in dermatology, to non-specialist health workers from peripheral centers, as part of the activities of the service. With an average of 14 cases per month, all CDPS used the telemedicine system for dermatology, some more than others. The system was reliable and effective for managing the vast majority of CDPS patients. This system is probably under-used by paramedical staff. Indeed the majority of requests come from physicians in peripheral centers, whereas the permanence of care is mainly represented by nurses in certain areas. The main weak points were the lack of information on patient's treatment follow-up and on clinical observations as well as the large number of photographs of insufficient quality.

### The Limits of the Study

Dermatology has been available since the launching of the system in 2001. Between 2001 and 2016, there has been an overall trend toward an increase in the use of teleservice in dermatology with a total of 1,790 applications and 112 requests per year in average. Dermatology is the most requested specialty since 2003 (radiology requests excluded). However, the study focused on a 18 months period. One reason was that the system has not been designed to extract data easily and it could have been challenging to study in details all the indicators of the MAST. Also, the retrospective nature generates biases imposing that lead us to be cautious about the interpretation of the results of this study. In fact, the feedback survey was not filled out prospectively after each request. Physicians and nurses working in CDPS in 2017 were contacted to answer the evaluation survey in order to limit the memory bias.

An objective method of evaluating changes in practice after regular use of the system would have made it possible to evaluate the evolution of referrers' knowledge of dermatology (diagnostic concordance) and the quality of the requests. Such a study was hardly feasible in the face of the frequent turn over in teams working in CDPS. The feedback survey response rate of 38.8% remained low. The Médecins Sans Frontières telemedicine system, which has been operating for 7 years, has been regularly evaluated by feedback surveys from users with comparable rate around 30% (1, 2).

### A Reliable, Efficient, Accessible, and Secure System Responding to the Needs of the Territory Marked by a Lack of Doctors

#### Needs

With two times less generalists and four times less specialists the medical density in French Guiana is lower than the metropolis and is concentrated on the coast. General practitioners would need the advice of a dermatologist in 25% of consultations for a dermatological reason^1^. Skin diseases are a very common reason for consultation in tropical settings and the infectious dermatoses play an important role as underlined by our study (46% of the cases). These often involve common pyoderma-like infectious diseases. However, neglected tropical diseases with specific management must also be detected without delay, such as leishmaniasis, leprosy, and Buruli ulcer. Neglected tropical diseases account for 11% of diagnoses in our study. The diagnosis and the fast management of some of these pathologies is an issue for the patient for whom an early diagnosis will limit complications and disabilities and a public health issue to reduce morbidity and limit transmission. There is little telemedicine experience for neglected tropical diseases in the literature. Brazilian study assesses the relevance of telemedicine for confirming a diagnosis of distant leprosy compared to a face-to-face examination. The specificity was 78% suggesting that telemedicine could be a useful method of diagnostic assistance to control this pathology, which remains a public health problem in some countries (3).

#### A Reliable, Efficient, Accessible, and Secure System

All requests received a response from specialists attesting to the reliability of the system. The median response time was 1 day and 12 min. In France the average time for appointments with a dermatologist is estimated at 64 days^2^. A telemedicine experiment in Hauts-de-France for the detection of cutaneous tumors linking 11 liberal dermatologists and 91 general practitioners had a response time of 3.5 days^3^. The Médecins Sans Frontières telemedicine system with 11 dermatologists around the world had a median time of 10.2 h (1). In Brazil the state of Minas Gerais telemedicine system (Telehealth Network of Minas Gerais TNMG) providing support to small municipalities had a response time for all specialties from 12 to 48 h (4). The French Guiana telemedicine system for dermatology therefore has a response time that seems acceptable. This system is easily accessible in the CPDS, all equipped with a computer with digital camera. On the other hand, this network is secure and facilitates the sharing of medical information.

#### Get Expert Center Membership to Support Front-Line Health Workers

The current system relies on the participation of dermatologists from the Cayenne Hospital Department. Its interest based on the territorial expertise is to make sure of the good knowledge of local pathologies (with a black skin experience), the therapeutic possibilities in the field and thus to bring an adapted answer. Dermatologists have integrated telemedicine into their practice without financial incentive or strengthening of teams. The use of the regional network of specialists has might has helped to get appropriate answers to the resources as 60% were very well-adapted and 76% of cases didn't require any additional exams and 57% could be treated with local treatment.

#### The Majority of Telexpertise Cases Are Managed Locally at CDPS, Thus Limiting Transfers to the Specialized Center

Our study showed a strong participation of the Maripasoula site (26%), this center was also the most demanding center for all specialties since 2014. This is probably related to the fact that it is the most important center in terms of in-patient consultations. The number of teleexpertise represents ~2.5% compared to the total number of consultations of the center including every medical specialty in 2016.

The small number of patients (8%) who are urgently evacuated or referred for specialized consultation or hospitalization at the Cayenne Hospital Center, suggests that the system has prevented a certain number of face to face consultation (92%) and therefore reduced costs related to these and associated transportation. A Brazilian study estimated at 81% the number of specialized consultations avoided thanks to telemedicine (5). In a literature review of 12 studies using teledermatology, the percentage of avoided travel was 43% (6). A medico-economic study conducted between 2001 and 2010 showed that French Guiana telemedicine network was cost-effective. Technological investments and operating costs were amortized by the savings made on lower transport costs, medical evacuations, hospitalizations, and consultations. Thus, out of 2,121 requests made between 2001 and 2010 it was estimated that the total cost in the absence of telemedicine would have been 1,538,930 Euros in travel expenses, consultations, and hospitalizations. Requests for radiology between 2006 and 2010 resulted in a total saving estimated at 354,000 euros by avoiding 59 medical evacuations to Fort-de-France (Martinique). The total economy was therefore estimated at EUR 1,892,930, while the total budget allocated to the system since 2001 was EUR 1,696,000. The new funding prospects for telemedicine could promote its development. In fact, in 2018, it entered the common law of medical practices and is now reimbursed by the health insurance^4^.

#### Satisfied and Better Trained Users to Manage Dermatoses in Tropical Areas

The feedback survey sent to doctors and nurses working in the CDPS centers in 2017 evaluated the satisfaction of the Guyanese teledermatology system at 7/10. The survey also highlighted an educational benefit for 93% of respondents. The interest of teledermatology in the continuing education of non-specialist doctors has been shown in several studies. In Burkina Faso, an assessment of diagnostic concordance between referrers and specialists over time showed that regular use of telemedicine in dermatology improved the knowledge of users (7).

### System Weaknesses and Improvement Pathways

#### Patient Follow-Up Is Rarely Done After Receiving the Specialist's Answer

One of the reason given for 40% of referrers was an ignorance of the need to provide follow-up. When a specialist sends a response, having a follow-up on the relevance, its applicability in the field, the future of the patient and the effectiveness of the treatment are important points. Follow-up does not only maintain the motivation of specialists to respond to requests, it would allow quality and adapted responses. It is also important for evaluating and monitoring the system's quality. A monitoring and evaluation program is not available in all telemedicine systems. The telemedicine system of the state of Minas Gerais in Brazil has developed a quality program including regular assessments of specialist responses (8). A survey was sent to referrers systematically after each expertise and included three questions: Did the consultation avoid addressing the patient in consultation? Did the remote consultation answer your question? How satisfied are you with the current system? Surveys of satisfaction sent systematically allow to ensure system quality since each unsatisfied answer entails a reason evaluation and the installation of correction and adaptation system. In our study, the medical turnover as well as the initial absence of computerized patient medical records on the system have probably been an obstacle to patient follow-up. Quality program and trainings on telemedicine might have an interest to focus on this point.

#### The Relevance of the Answers Depends on the Quality's Requests

Our study highlights the importance of the quality of the demand, whose relevance of the answer depends. A request for clarification or technical advice on the taking of photographs by the specialist concerned one out of four requests. Also, 40% of the photographs were judged of medium or bad quality. The diagnosis of the specialist was judged as not certain in 38% of the cases. These results reinforce the importance of completing the request forms exhaustively and providing good quality images. To date, the use of telemedicine is possible in each CDPS center, with general training at the Cayenne Hospital available since 2008 as well as an annual visit of all sites once a year by a physician and a computer scientist. However, the beneficiaries do not have any initial training and good practice recommendations for dermatology. Training is possible annually in the service but not mandatory. Physicians on CDPS sometimes come from outside the territory for a limited time and cannot attend. An online training system could be useful for users with significant turnover. Providing such training could increase the visibility of the system for more optimal use.

#### A System Barely Used by Non-medical Health Workers

Telemedicine system for dermatology was mainly used by physicians (70% of requests) and to a lesser extent by nurses (18%). Of the 16 CDPS in French Guiana, eight centers are staffed only by nurses. Experiences in similar contexts where telemedicine is used to obtain specialist advice in front-line health centers show that requests can be sent by nurses. In Brazil, for example, 50% of TNMG requests are made by nurses (5). The telemedicine system in French Guiana is probably underused by paramedical teams.

#### Technological Limits

The technology used in French Guiana requires the downloading of a software on a computer. Consulting the telexpertise forms must be done exclusively in the Cayenne dermatology unit and case management is not possible at a distance from a personal computer or a mobile phone. The exchanges are between centers and well-defined specialists who have the software. In the literature, publications on telemedicine are increasingly interested in mobile technologies that open new avenues for improving the supply of health care. There are many mobile apps especially for dermatology. Some may be useful for training. In 2017, there were more than 500 mobile applications in dermatology with a significant growth in telemedicine applications (9). One example is the SkinApp application developed to improve the management of dermatological diseases in front-line health centers. This application aims to present the most common pathologies in tropical context as well as neglected tropical dermatoses that require specific care and without delay. The increasing use of smartphones and the considerable improvement of their image resolution is no longer a limit to their use in teledermatology. Moreover, in France, three-quarters of physicians own a smartphone and more than 9/10 use it for professional purposes^5^ and some applications have shown a good sensitivity (98%) in dermatological telexpertise (10). However, many areas in French Guiana still have very poor network and mobile internet coverage^6^ which require tools adapted to these condition with low connectivity. Protection of personal data, health data and confidentiality are the main issues related to the development of such applications.

## Conclusion

Our study shows that teledermatology is useful in the context of French Guiana. First for patients who benefit from the expertise of a dermatologist without delay or displacement. Then for physicians and nurses who have direct access to specialists and benefit from a quick and adapted specialized advice, helping in their medical education. Finally for the healthcare system since the activity of specialists is part of the dermatology department and the technological investments are offset by the reduction of medical evacuations, hospitalizations, and consultations. The vast majority of CDPS patients discussed with the specialist were being treated via telemedicine. Efforts remain to be made on the quality of the requests sent and on patients follow-up, probably via improving users training in telemedicine particularly in the dermatology field.

## Data Availability Statement

The datasets generated for this study are available on request to the corresponding author.

## Ethics Statement

Ethics permission was not required, because the work was a retrospective chart review conducted by the hospital staff.

## Author Contributions

A-LM: article writing. PC and RB: rereading. SD: supervising and rereading.

### Conflict of Interest

The authors declare that the research was conducted in the absence of any commercial or financial relationships that could be construed as a potential conflict of interest.

## Footnotes

^1^Stéphanie Avogadro-Leroy, “Pathologies cutanées en médecine générale : une étude quantitative en Haute-Normandie,” December 2012. https://dumas.ccsd.cnrs.fr/dumas-00768337/document.

^2^Médecins : Le Délai d'attente Pour Obtenir Un Rendez-Vous Région Par Région,” http://www.lefigaro.fr/conjoncture/2017/03/23/20002-20170323ARTFIG00004-dentiste-orl-opthalmologue-decouvrez-le-delai-d-attente-par-region.php.

^3^“Télédermatologie,” URPS Médecin libéraux Hauts de France http://www.urpsml-hdf.fr/teledermatologie/.

^4^“Coup d'envoi du remboursement de la télémédecine : 15 septembre 2018,” Medicitus https://www.medicitus.com/coup-denvoi-du-remboursement-de-la-telemedecine-15-septembre-2018.

^5^Livre blanc santé connectée, January 2015, https://www.conseil-national.medecin.fr/node/1558.

^6^“Mon Réseau Mobile|Arcep,” https://www.monreseaumobile.fr.
